# Nuclear deformation mediates liver cell mechanosensing in cirrhosis

**DOI:** 10.1016/j.jhepr.2020.100145

**Published:** 2020-07-17

**Authors:** Sergi Guixé-Muntet, Martí Ortega-Ribera, Cong Wang, Sonia Selicean, Ion Andreu, Jenny Z. Kechagia, Constantino Fondevila, Pere Roca-Cusachs, Jean-François Dufour, Jaime Bosch, Annalisa Berzigotti, Jordi Gracia-Sancho

**Affiliations:** 1 Department of Biomedical Research, University of Bern, Bern, Switzerland; 2 Liver Vascular Biology Research Group, Hepatic Hemodynamic Laboratory, IDIBAPS Biomedical Research Institute, CIBEREHD, Barcelona, Spain; 3 Institute for Bioengineering of Catalonia, Barcelona Institute of Technology, University of Barcelona, Barcelona, Spain; 4 Liver Surgery, Hospital Clinic de Barcelona, CIBEREHD, Barcelona, Spain; 5 Hepatology, University Clinic for Visceral Surgery and Medicine, Inselspital, Bern, Switzerland

**Keywords:** Chronic liver disease, Hepatocyte, HSC, LSEC, Stiffness, ACLD, advanced chronic liver disease, Cd, cytoskeleton disruptor, ECM, extracellular matrix, eNOS, endothelial nitric oxide synthase, HNF4α, hepatocyte nuclear factor 4α, HSC, hepatic stellate cell, KASH, Klarsicht/abnormal nuclear anchorage-1/Syne homology, Lamb1, laminin b1, LSEC, liver sinusoidal endothelial cell, DN-KASH, dominant negative nesprin peptide containing a KASH domain, TAA, thioacetamide, α-SMA, α-smooth muscle actin

## Abstract

**Background & Aims:**

Liver stiffness is increased in advanced chronic liver disease (ACLD) and accurately predicts prognosis in this population. Recent data suggest that extracellular matrix stiffness *per se* may modulate the phenotype of liver cells. We aimed at investigating the effect of matrix stiffness on the phenotype of liver cells of rats with cirrhosis, assessing its influence on their response to antifibrotic strategies and evaluating associated molecular mechanisms.

**Methods:**

Hepatocytes, hepatic stellate cells, and liver sinusoidal endothelial cells were isolated from healthy rats or rats with cirrhosis (carbon tetrachloride or thioacetamide), and cultured on polyacrylamide gels with different physiologically relevant stiffness for 72 h.

**Results:**

All cell types of rats with cirrhosis cultured at low stiffness showed a significant phenotype amelioration *vs.* rigid matrix (assessed by quantitative morphology, mRNA expression, protein synthesis, and electron microscopy imaging). Additionally, stiffness modified the antifibrotic effects of liraglutide in stellate cells of rats with cirrhosis. Finally, evaluation of nuclear morphology revealed that high stiffness induced nuclei deformation in all cell types, an observation confirmed in cells from human livers. Disconnecting the nucleus from the cytoskeleton by cytoskeleton disruption or a defective form of nesprin 1 significantly recovered spherical nuclear shape and quiescent phenotype of cells.

**Conclusions:**

The environment's stiffness *per se* modulates the phenotype of healthy rats and liver cells of rats with cirrhosis by altering the nuclear morphology through cytoskeleton-derived mechanical forces. The reversibility of this mechanism suggests that targeting the stiffness-mediated intracellular mechanical tensions may represent a novel therapeutic strategy for ACLD.

**Lay summary:**

During cirrhosis, the liver becomes scarred, stiff, and unable to perform its normal functions efficiently. In this study, we demonstrated that cells from diseased (stiff) livers recovered their functionality when placed in a soft environment (as that of a healthy liver). Furthermore, treatments aimed at *tricking* liver cells into believing they are in a healthy, soft liver improved their function and could potentially contribute to treat cirrhosis.

## Highlights

• Low stiffness directly improves the phenotype of hepatocytes, hepatic stellate cells, and liver sinusoidal endothelial cells.
• Stiffness modulates sinusoidal crosstalk in advanced chronic liver disease (ACLD).
• Stiffness may determine the efficacy of antifibrotic strategies.
• Mechanical forces applied to the nucleus through the LINC complex mediate the effects of stiffness on liver cell phenotype.

## Graphical abstract

**Figure:**
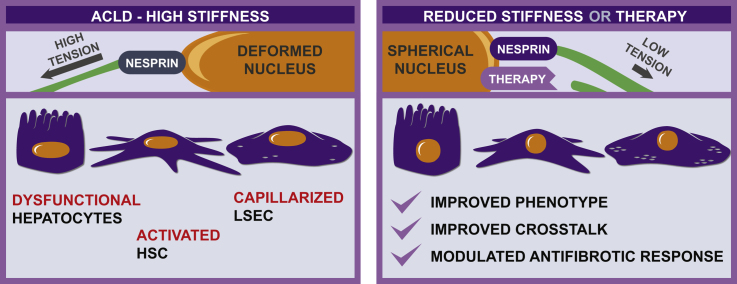

## Introduction

Liver fibrosis results from excessive extracellular matrix (ECM) deposition and formation of scar tissue in response to persistent liver injury (*e.g.* prolonged excessive alcohol consumption, NASH, or chronic viral hepatitis). Fibrosis and associated tissue remodelling modify the mechanical properties of the liver, leading to a shift from an elastic, soft organ, well compliant to increases in blood flow, to a stiff organ, with high resistance to flow. As such, fibrosis in combination with liver microvascular dysfunction is the primary factor driving the development of portal hypertension in advanced chronic liver disease (ACLD).^1^

Recent data suggest that the phenotype of a cell could be modulated by the structural/mechanical properties of the surrounding ECM.[2], [3], [4] Specifically, observations in healthy hepatocytes and hepatic stellate cells (HSCs) indicate that increased stiffness of the culture substrate induces activation of these cells,[5], [6], [7], [8] whilst reseeding these *in vitro*-activated HSCs on soft matrices partially reverts their activation.^9^^,^^10^ Nevertheless, the effects of ECM stiffness on the phenotype of primary cells isolated from livers of rats with cirrhosis and its underlying mechanisms remain unknown. These may be highly relevant as hepatic cells in an organ with cirrhosis (stiff) would permanently receive activating biomechanical signals that may prevent fibrosis regression even after successfully treating the cause of the liver disease, suggesting that stiffness mechanotransduction may represent a novel therapeutic target. This hypothesis is supported by clinical observations; patients with ACLD in the same stage of the disease can have different values of liver stiffness, and in these settings higher liver stiffness predicts a higher probability of clinical complications and thus a worse prognosis.^11^^,^^12^ Even in the scenario of decompensated cirrhosis, when severe portal hypertension and very advanced fibrosis are always found, liver stiffness remains associated with mortality independently of model for end-stage liver disease score.^13^

With this background, we herein aimed at characterising the effects of matrix stiffness on the modulation of liver cell phenotype of rats with cirrhosis as well as determining the underlying molecular mechanisms.

## Materials and methods

### Animals

Male Sprague Dawley rats were kept at the animal facilities of the University of Bern, whilst male Wistar Han rats were used at the animal facilities of the University of Barcelona Medical School. All animals were maintained in controlled environmental conditions with 12 h light-dark cycles, and fed *ad libitum* with water and standard rodent food. All experiments were approved by the Bern Cantonal Ethics Committee and the Laboratory Animal Care and Use Committee of the University of Barcelona, and were conducted in accordance with the European Community guidelines for the protection of animals used for experimental and other scientific purposes (EEC Directive 86/609).

### Induction of liver cirrhosis

For the carbon tetrachloride (CCl_4_) model, cirrhosis was induced in male Wistar rats weighing 100 g by chronic CCl_4_ inhalation (thrice per week) whilst receiving 0.3 g/L phenobarbital in the drinking water. Rats were considered with cirrhosis after development of ascites, which takes 14–16 weeks, approximately.

For the thioacetamide (TAA) model, male Sprague Dawley rats weighing 250 g were injected with 250 mg/kg TAA i.p. twice a week for 12 weeks.

### Human samples

Human tissues were obtained from remnant pieces from partial hepatectomy to excise tumour metastasis from colon carcinoma (for healthy tissues) or from explanted livers from patients with cirrhosis receiving orthotopic liver transplantation (NASH or chronic ethanol aetiology). HSCs were isolated from 3 different livers of patients with cirrhosis. The Ethics Committee of the Hospital Clínic de Barcelona approved the experimental protocol (HCB/2015/0624), and patients gave their signed informed consent.

Immortalised human-activated HSC LX-2 cells (kindly provided by Dr Bataller) were used for transfection and toxicity experiments. Results using LX-2 cells were derived from at least 3 different batches per experimental condition.

### Isolation of hepatic cells and treatments

Isolation of primary cells was performed as previously described.^14^ Please see the supplementary information for further details regarding cell isolation and pharmacological treatments.

### Preparation of matrix gels with different stiffnesses

Collagen-coated polyacrylamide gels were prepared as described in the supplementary section.^15^

The stiffnesses of the gels were established at 0.5, 13.5, and 30 kPa (Young's modulus),^6^^,^^16^ which correlate with clinical measurements of liver stiffnesses (shear modulus) in healthy livers, livers with advanced fibrosis, and decompensated livers of patients with cirrhosis, respectively.^17^

### Co-culture experiments

HSCs and liver sinusoidal endothelial cells (LSECs) were freshly isolated from rats with cirrhosis and directly plated either on gels with 0.5 or 30 kPa, or on transwell inserts with adjustable height (Thermo Fisher). After 12 h of monoculture, cells were washed with PBS and co-culture was started by placing the HSC-containing inserts on top of the LSEC-containing gels and vice versa (with inserts set at their medium hanging position), and allowed to communicate for additional 36 h. The mRNA expression of phenotypic markers was assessed in the cells contained in the insert (which were not in direct contact with the gels).

### EGFP-nesprin 1-*KASH* plasmid transfection

Human HSCs (LX-2 and primary HSCs from patients with cirrhosis) were transfected with the dominant-negative plasmid EGFP-nesprin 1-*KASH* (kindly provided by Catherine Shanahan)^18^ or EGFP-empty plasmid using a neon transfection device (Thermo Fisher) as described previously.^4^ Efficiency of transfection and transfection controls was assessed by EGFP fluorescence. Then, 24 h after the transfection, cells were cultured on collagen-coated polyacrylamide gels for 3 days.

### Hepatocyte function

Hepatocytes were washed twice with PBS and culture medium was changed every 24 h. Synthesis of urea and albumin was evaluated at the Zentrum für Labormedizin (Inselspital, University Hospital of Bern) in the supernatant corresponding to the time range of 48–72 h and normalised to the number of cells of the corresponding well.

### Bright field morphology analysis

Four images per well (100× magnification) were taken using a standard phase contrast microscope. Differences in cell morphology were quantified as described in the supplementary information.

### Scanning electron microscopy

LSEC fenestration was assessed by scanning electron microscopy.^14^ LSECs were fixed overnight with 2% glutaraldehyde dissolved in 0.1 M cacodylate buffer (pH 7.4). After 3 washes with cacodylate buffer for 5 min, the cover glasses were incubated for 1 h with 1% tannic acid and subsequently incubated with 2% osmic acid for 2 h. The samples were dehydrated with an ethanol battery (50%, 70%, 90%, 95%, and 100%), critical-point dried with carbon dioxide, sputter coated with gold, and examined by scanning electron microscopy.

### Immunofluorescence and confocal microscopy

Please see the Supplementary methods section for details about processing of the samples and imaging, including nuclear deformation analyses.

### Evaluation of nitric oxide levels

LSECs were incubated with Roswell Park Memorial Institute's medium without phenol red containing 3 ng/ml DAPI (Thermo Fisher) and 5 μg/ml diaminofluorescein diacetate (Thermo Fisher) for 20 min at 37°C. After 2 washes with PBS, 6 images per cover glass and channel (blue, 405 nm; green, 488 nm) were taken. The same settings were used for gathering and quantification of all images. Intensities were normalised to the number of cells per field.^19^

### Real-time PCR

Real-time PCR was performed using TaqMan probes, specified in the Supplementary methods.

### Statistical analyses

Statistical analyses were performed using the IBM SPSS 23 statistics software for Windows. Normality of sample distribution was assessed using the Kolmogorov-Smirnov test. For samples fitting a normal distribution, means were compared by the Student *t* test (2 samples) or ANOVA (>2 samples) followed by the Tukey *post hoc* analysis. Otherwise, means were compared using the non-parametric Kruskal-Wallis test followed by the Mann-Whitney *U* test. Differences were considered significant at *p* <0.05.

## Results

### High matrix stiffness induces the activation of healthy liver cells

The effect of matrix stiffness on the phenotype of healthy hepatic cells was evaluated in freshly isolated hepatocytes, HSCs, and LSECs from healthy rat livers, and plated on polyacrylamide gels with increasing stiffness. Hepatocytes, HSCs, and LSECs plated at higher stiffness (30 kPa) became apparently larger and flatter than on physiological stiffness (0.5 kPa). Moreover, quantification of texture descriptors confirmed a shift in cell morphology at 72 h of culture (Fig. S1A–C, top panels).

These differences in cell morphology were accompanied by alterations in phenotype markers. Healthy hepatocytes cultured for 72 h at 30 kPa had impaired functionality, as shown by reduced hepatocyte nuclear factor 4α (HNF4α) and albumin mRNA expression (Fig. S1A, middle). Alterations at the mRNA level translated into decreased hepatocyte function as demonstrated by significantly reduced release of urea and albumin to the culture media in high stiffness conditions (Fig. S1A, bottom).

Culture of quiescent HSCs on high stiffness (30 kPa) gels did not change the mRNA expression of the activation markers α-smooth muscle actin (α-SMA) and collagen I (Fig. S1B, middle). However, we observed a post-translational effect of high stiffness on HSCs, characterised by a reorganisation of the α-SMA protein, occupying a greater area and arranged in stress fibres, in contrast to the diffuse fluorescence observed at low matrix stiffness (Fig. S1B, bottom).

Finally, 72 h culture on different stiffnesses also modified the phenotype of LSECs. When cultured on high stiffness surfaces, LSECs displayed higher levels of the capillarisation marker laminin b1 (Lamb1) at the mRNA level. In addition, LSECs cultured on high stiffness gels showed an upregulation of endothelial nitric oxide synthase (eNOS) mRNA (Fig. S1C, middle), similar to what has been described previously in the liver of rats with cirrhosis.^20^ However, increased eNOS gene expression was not accompanied by an improved phenotype, but by reduced nitric oxide synthesis, increased number of attached cells, and reduced fenestration (Fig. S1C, bottom) in high *vs.* low stiffness matrices.

Collectively, our observations confirm previous reports showing activation of HSCs and hepatocytes in response to high stiffness, and reveal that LSECs become capillarised when cultured in high stiffness matrices.

### Culture at low stiffness (mimicking healthy conditions) improves the phenotype of liver cells of rats with cirrhosis

We evaluated the effects of substrate stiffness in cells isolated from CCl_4_-exposed rats with cirrhosis, cultured at decreasing stiffness for 72 h.

**Fig. 1:**
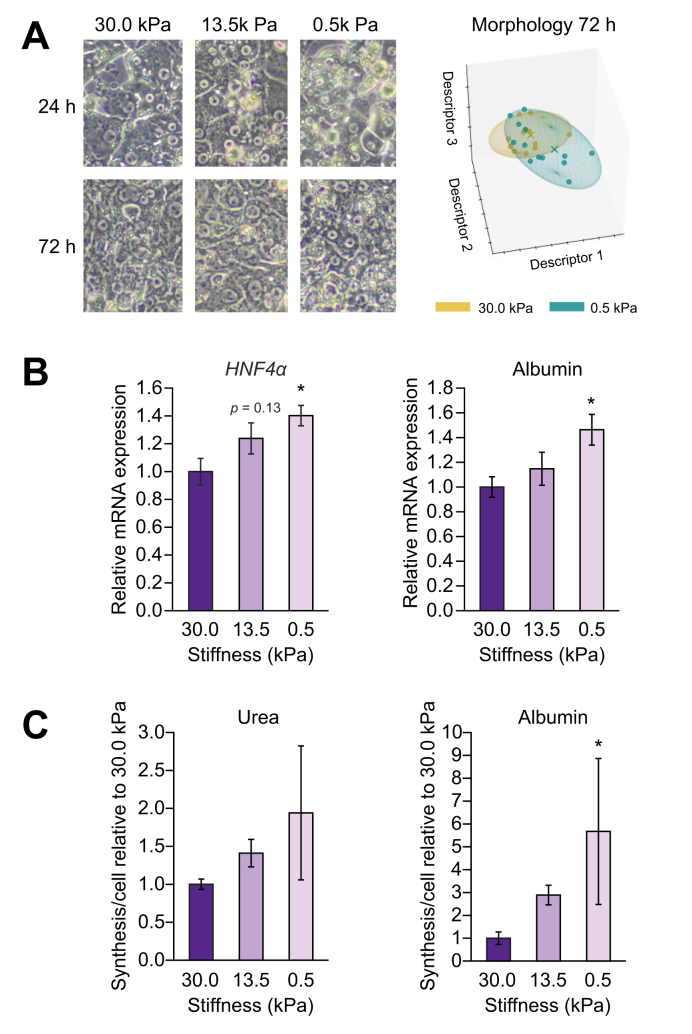
Low (healthy) stiffness promotes improvement of hepatocytes of rats with cirrhosis. Hepatocytes from CCl_4_-exposed rats with cirrhosis were cultured for 72 h on polyacrylamide gels with decreasing stiffness. (A) Bright field images and corresponding morphology quantification. (B) mRNA expression of the phenotype markers HNF4α and albumin. (C) Levels of urea and albumin released during the last 24 h of culture. Data derived from 4 independent experiments and are expressed as mean ± SEM. For each experiment, sample distributions were assessed for normality (Kolmogorov-Smirnov test) and homoscedasticity (Levene's test). ANOVA was performed in homoscedastic groups following a normal distribution, followed by Tukey's *post hoc* test. Otherwise, the non-parametric Kruskal-Wallis was performed, followed by the Mann-Whitney *U* test. ∗*p* <0.05 *vs.* 30 kPa; *p* >0.2 if not specified. HNF4α, hepatocyte nuclear factor 4α.

**Fig. 2:**
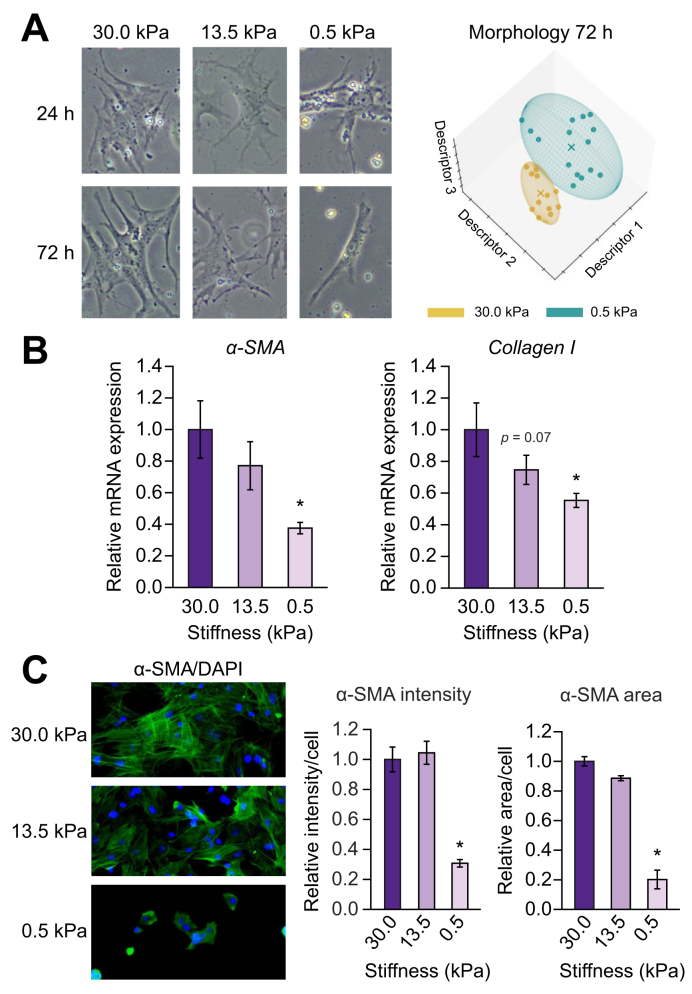
Low (healthy) stiffness promotes improvement of HSCs of rats with cirrhosis. HSCs from CCl_4_-exposed rats with cirrhosis were cultured for 72 h on polyacrylamide gels with decreasing stiffness. (A) Bright field images and corresponding morphology quantification. (B) mRNA expression of the phenotype markers α-SMA and collagen I. (C) Immunofluorescence of α-SMA (green) and corresponding quantification of intensity and area. Nuclei were stained with DAPI (blue). Data derived from 4 independent experiments and are expressed as mean ± SEM. For each experiment, sample distributions were assessed for normality (Kolmogorov-Smirnov test) and homoscedasticity (Levene's test). ANOVA was performed in homoscedastic groups following a normal distribution, followed by Tukey's *post hoc* test. Otherwise, the non-parametric Kruskal-Wallis was performed, followed by Mann-Whitney *U* test. ∗*p* <0.05; *p* >0.2 if not specified. HSC, hepatic stellate cell; α-SMA, α-smooth muscle actin.

**Fig. 3:**
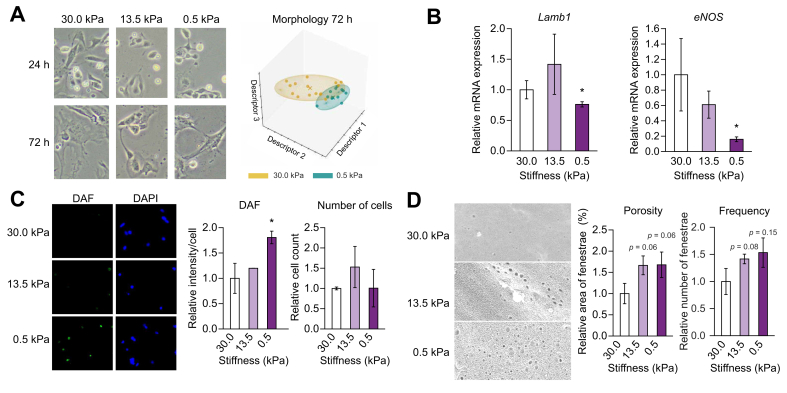
Low (healthy) stiffness promotes improvement of LSECs of rats with cirrhosis. LSECs from CCl_4_-exposed rats with cirrhosis were cultured for 72 h on polyacrylamide gels with decreasing stiffness. (A) Bright field images and corresponding morphology quantification. (B) mRNA expression of the phenotype markers Lamb1 and eNOS. (C) Nitric oxide synthesis (diaminofluorescein diacetate; green), corresponding quantification of intensity and number of cells (number of nuclei). Nuclei were stained with DAPI (blue). (D) Scanning electron microscopy images (5,000× magnification) and fenestrae quantification (porosity and frequency). Data derived from 4 independent experiments and are expressed as mean ± SEM. For each experiment, sample distributions were assessed for normality (Kolmogorov-Smirnov test) and homoscedasticity (Levene's test). ANOVA was performed in homoscedastic groups following a normal distribution, followed by the Tukey's *post hoc* test. Otherwise, the non-parametric Kruskal-Wallis was performed, followed by Mann-Whitney *U* test. ∗*p* <0.05; *p* >0.2 if not specified. eNOS, endothelial nitric oxide synthase; Lamb1, laminin b1; LSEC, liver sinusoidal endothelial cell.

Similar to cells isolated from control (without cirrhosis) rats, the phenotype of liver cells of rats with cirrhosis was also affected by stiffness. Hepatocytes, HSCs, and LSECs isolated from rats with cirrhosis cultured on low, healthy stiffness (0.5 kPa) displayed morphological changes when compared with cells cultured on stiffer substrates (Figs. 1A, 2A, and 3A, respectively). These morphological changes were accompanied by an improvement in their phenotype. Indeed, hepatocytes isolated from livers of rats with cirrhosis cultured for 72 h on low stiffness ameliorated their functionality with respect to those cultured at higher stiffness, as shown by both mRNA markers (Fig. 1B) and *in vitro* urea and albumin synthesis and release (Fig. 1C).

Low stiffness surfaces ameliorated HSC over-activation, displaying lower mRNA levels of the activation markers α-SMA and collagen I compared with stiffness of rats with cirrhosis (Fig. 2B), without affecting the expression of the quiescence markers Hhip or Lrat, nor cell viability (data not shown). The beneficial effects of low stiffness were also seen at a post-transcriptional level as diminished α-SMA protein intensity, area, and rearrangement of stress fibres (Fig. 2C).

Similarly, LSECs isolated from livers of rats with cirrhosis and cultured on low stiffness (0.5 kPa) exhibited a decreased expression of the capillarisation marker Lamb1. Moreover, whilst their eNOS mRNA expression decreased (Fig. 3B), their NO bioavailability improved (Fig. 3C). Additionally, low stiffness induced an increase in the frequency and porosity of fenestrae (Fig. 3D).

Improvement of the phenotype in low stiffness conditions was additionally validated in primary HSCs isolated from TAA-injected rats with cirrhosis. Also in these experiments, HSC activation markers α-SMA and collagen I were significantly decreased in cells cultured on low stiffness substrates compared with stiffness conditions of rats with cirrhosis (Fig. S2).

### Stiffness modulates sinusoidal cell-to-cell communication

**Fig. 4:**
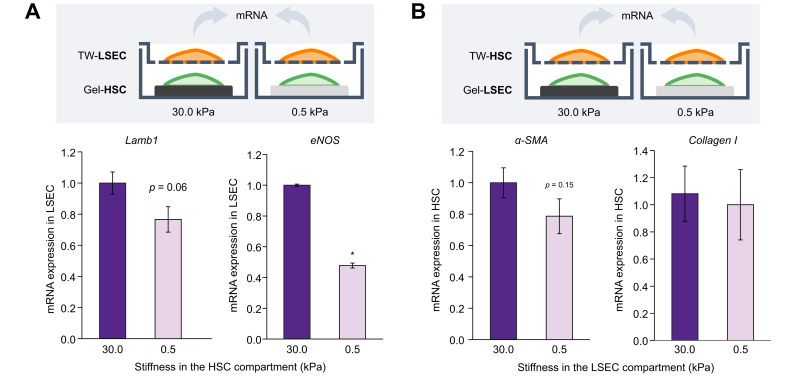
Stiffness modulates the crosstalk between sinusoidal cells of rats with cirrhosis. LSECs and HSCs isolated from rats with cirrhosis were co-cultured for 36 h on polyacrylamide gels or TWs. (A) Analysis of LSEC capillarisation markers (Lamb1 and eNOS) after co-culture with HSCs cultured on stiff (30 kPa) or soft (0.5 kPa) matrices. (B) Analysis of HSC activation markers (α-SMA and collagen I) after co-culture with LSECs on polyacrylamide tuned gels. Data derived from 3 independent experiments and are expressed as mean ± SEM. Groups were compared with Student *t* test. ∗*p* <0.05 *vs.* 30 kPa; *p* >0.2 if not specified. eNOS, endothelial nitric oxide synthase; HSC, hepatic stellate cell; Lamb1, laminin b1; LSEC, liver sinusoidal endothelial cell; TW, transwell; α-SMA, α-smooth muscle actin.

In addition to the direct effect on cellular function, we assessed whether stiffness may influence crosstalk between sinusoidal cells of rats with cirrhosis. For this, we isolated HSCs and LSECs from rats with cirrhosis and plated 1 cell type on stiffness-modulated gels at the bottom of the well, which was allowed to communicate paracrinally with the other cell type, attached on the permeable membrane of a transwell (Fig. 4, top). HSCs of rats with cirrhosis improved by a healthier stiffness exerted beneficial effects on LSECs of rats with cirrhosis (Fig. 4A), reducing the expression of laminin and significantly ameliorating eNOS mRNA expression. In addition, direct contact of LSECs of rats with cirrhosis with soft or stiff matrices had no clear paracrine effect on HSC activation of rats with cirrhosis (Fig. 4B), although there was a trend for decreased α-SMA.

### Stiffness modulates the cellular response to antifibrotic drugs

**Fig. 5:**
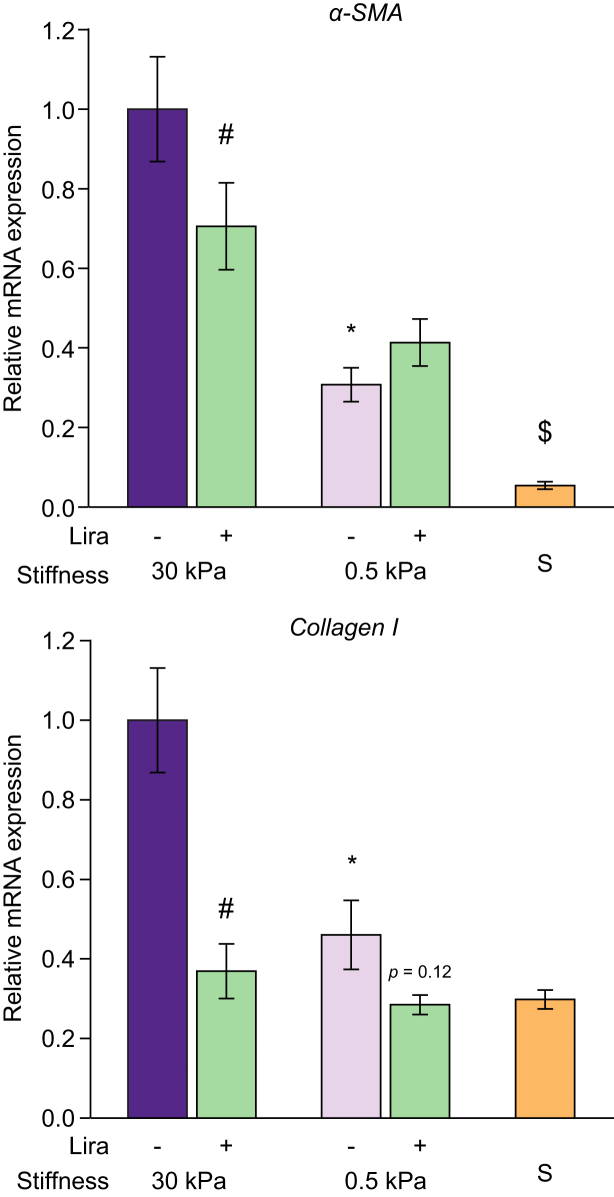
Stiffness modulates antifibrotic effect of liraglutide. HSCs from CCl_4_-exposed rats with cirrhosis were cultured for 72 h on gels with high (30 kPa) or low (0.5 kPa) stiffness in the presence of vehicle or liraglutide. Graphs correspond to relative mRNA expression of the activation markers α-SMA and collagen I. Data derived from 3 independent experiments and are expressed as mean ± SEM. Treatment with simvastatin (S) at 0.5 kPa was used as a positive control of further HSC deactivation. For each experiment, sample distributions were assessed for normality (Kolmogorov-Smirnov test) and homoscedasticity (Levene's test). ANOVA was performed in homoscedastic groups following a normal distribution, followed by Tukey's *post hoc* test. Otherwise, the non-parametric Kruskal-Wallis was performed, followed by Mann-Whitney *U* test. ∗*p* <0.05 *vs.* 30 kPa, ^†^*p* <0.05 *vs.* corresponding vehicle. ^‡^*p* <0.05 *vs.* 0.5 kPa + liraglutide. *p* >0.2 if not specified. HSC, hepatic stellate cell; α-SMA, α-smooth muscle actin.

CCl_4_-exposed HSCs of rats with cirrhosis cultured on high stiffness matrices and treated with 50 μM liraglutide (a drug with described antifibrotic effects)^21^ showed a significant decrease in the mRNA markers of HSC activation α-SMA and collagen I. This downregulation, however, was not observed in cells cultured on low stiffness matrices (Fig. 5), indicating that stiffness could influence the response to pharmacological antifibrotic strategies. In these experiments, 10 μM simvastatin was used as a positive control for HSC deactivation, showing that at 0.5 kPa α-SMA expression could be further reduced, supporting the idea that liraglutide was indeed less effective at 0.5 kPa and not just because α-SMA expression reached a minimum.

### Stiffness impairs nuclear sphericity of liver cells

**Fig. 6:**
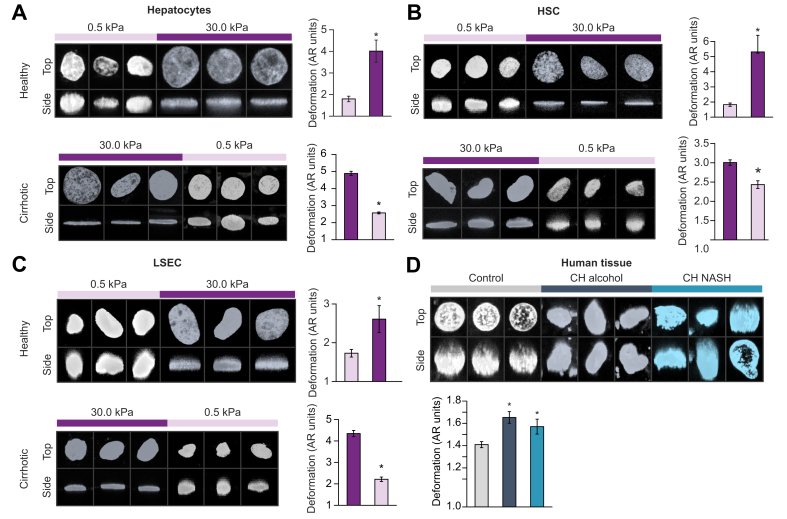
Stiffness determines deformation of the nucleus in liver cells. (A) Primary hepatocytes, (B) HSCs, and (C) LSECs were isolated from healthy or CCl_4_-exposed rats with cirrhosis (top and middle graphs, respectively) and cultured on gels with low (0.5 kPa) or high (30 kPa) for 72 h. Images of their nuclei were obtained with a confocal microscope, and their deformation was quantified. (D) Morphology of the nuclei of human liver cells *in situ* in healthy or tissue sections of patients with cirrhosis (either alcohol or NASH aetiologies). More than 100 nuclei were analysed for each experimental condition. Images were taken at 630× magnification. N = 3 samples per experimental condition. Data are expressed as mean ± SEM. For each experiment, sample distributions were assessed for normality (Kolmogorov-Smirnov test) and homoscedasticity (Levene's test). For experiments with 2 groups, these were compared with Student *t* test. For multiple comparisons, ANOVA was performed in homoscedastic groups following a normal distribution, followed by Tukey's *post hoc* test. Otherwise, the non-parametric Kruskal-Wallis was performed, followed by Mann-Whitney *U* test. ∗*p* <0.05; *p* >0.2 if not specified. AR, aspect ratio; CH, cirrhotic; HSC, hepatic stellate cell; LSEC, liver sinusoidal endothelial cell.

**Fig. 7:**
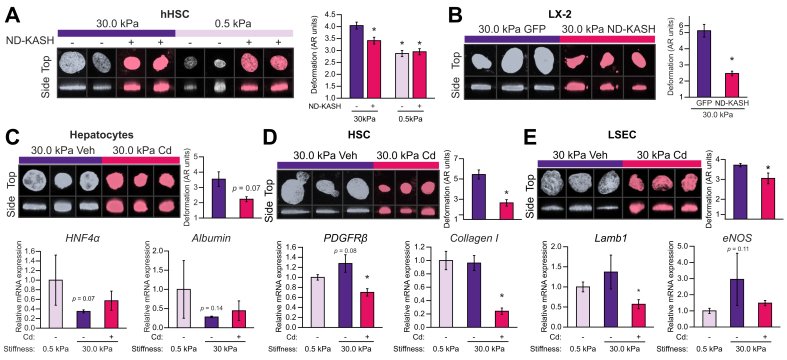
Uncoupling the nucleus from cytoskeletal tensions reverts stiffness effects. (A) Primary HSCs of patients with cirrhosis and (B) LX-2 were transfected with EGFP-nesprin 1-KASH (DN-KASH) or EGFP-empty (GFP) plasmid and cultured for 72 h on polyacrylamide matrices. (C–E) Primary rat liver cells were cultured at 30 kPa for 72 h and treated with Cd or Veh for the last 24 h. Nuclear morphology and corresponding mRNA expression of phenotype markers were assessed. More than 100 nuclei were analysed for each experimental condition. Images were taken at 630× magnification. N = 3 samples per experimental condition. Data are expressed as mean ± SEM. For each experiment, sample distributions were assessed for normality (Kolmogorov-Smirnov test) and homoscedasticity (Levene's test). For experiments with 2 groups, these were compared with Student *t* test. For multiple comparisons, ANOVA was performed in homoscedastic groups following a normal distribution, followed by Tukey's *post hoc* test. Otherwise, the non-parametric Kruskal-Wallis was performed, followed by Mann-Whitney *U* test. ∗*p* <0.05 *vs.* 30 kPa Veh; *p* >0.2 if not specified. Cd, cytoskeletal disruptor; eNOS, endothelial nitric oxide synthase; HNF4α, hepatocyte nuclear factor 4α; HSC, hepatic stellate cell; KASH, Klarsicht/abnormal nuclear anchorage-1/Syne homology; Lamb1, laminin b1; DN-KASH, negative dominant nesprin peptide containing a KASH domain; PDGFRb, platelet-derived growth factor receptor beta; Veh, vehicle.

Nuclear deformation in healthy hepatocytes, HSCs, and LSECs cultured on high stiffness matrices was significantly greater than on low stiffness gels (Fig. 6A–C, top panels). In addition, cells isolated from livers of rats with cirrhosis, which displayed a flat nucleus on 30 kPa gels, reversed their nuclear morphology to a spherical shape when cultured at 0.5 kPa (Fig. 6A–C, lower panels). Stiffness effects on nuclear shape were also validated in human samples; DAPI staining of human liver tissues revealed that cells residing in livers of patients with cirrhosis (from both ethanol or NASH aetiologies) disclosed higher nuclear deformation compared with cells from healthy liver tissue. The degree of nuclear deformation was similar in both groups of rats with cirrhosis (Fig. 6D). By contrast, HSCs isolated from human livers with cirrhosis and seeded on soft matrices displayed improved nuclear morphology compared with those plated on hard matrices (Fig. 7A).

### Phenotypical response to stiffness is mediated by the nucleus-cytoskeleton interaction

To study the link between matrix stiffness and nuclear shape, human HSCs (LX-2 and primary HSCs of patients with cirrhosis) were transfected with a vector coding for a dominant negative nesprin peptide, containing a KASH domain (DN-KASH), inhibiting the coupling of the nucleus with the cytoskeleton^4^^,^^18^ and plated on stiff matrices. EGFP was used as control peptide, and transfection efficiency was verified by EGFP fluorescence in transfected groups (Fig. S3). Both primary HSCs of patients with cirrhosis and LX-2 transfected with DN-KASH displayed less nuclear deformation compared with EGFP-transfected cells (Fig. 7A and B, respectively). Interestingly, the effects of DN-KASH were not observed in HSCs plated on soft matrices (Fig. 7A), suggesting that the effect of matrix stiffness on the nuclear morphology of hepatic cells is dependent on cytoskeletal tension.

To further assess whether mechanical deformation of the nucleus induced by increased matrix stiffness determines the phenotype of liver cells, we used a pharmacological approach to uncouple the nucleus from the cytoskeleton using cytoskeletal disruptors (Cd; cytochalasin D plus nocodazole) and analysed the effects on cell phenotype. Cd proved effective in uncoupling the nucleus under high stiffness conditions in LX-2 and primary rat liver cells, as observed by broken actin filaments (Fig. S4), and ameliorated nuclear sphericity (Fig. S5A; Fig. 7C–E, top panels) without signs of toxicity (evaluated by lactate dehydrogenase release; Fig. S5B). Disruption of the matrix-nucleus link with Cd translated into an improved phenotype of liver cells, despite being cultured on high stiffness matrices. Hepatocytes partly recovered the mRNA expression of albumin and HNF4α (Fig. 7C); HSCs exhibited a reduction in the activation markers platelet-derived growth factor receptor beta and collagen I (Fig. 7D), and LSEC phenotype improved as demonstrated by reduced Lamb1 and eNOS mRNA expression (Fig. 7E) and a dramatic increase in the area and number of fenestrae (Fig. S6).

## Discussion

With 844 million cases and 2 million deaths per year, chronic liver disease represents 1 of the major public health problems worldwide.^22^^,^^23^ As morbidity and mortality mostly concern patients with ACLD, there is a need for therapeutic strategies aiming at improving the disease in this late stage.^24^

Mechanical forces, including ECM stiffness or mechanical stretch, can regulate the cellular phenotype.^5^^,^^6^^,^[25], [26], [27], [28] Indeed, cells attached to any ECM exert actomyosin forces on it to test its rigidity (stiffness sensing). Consequently, cytoskeletal forces are transmitted to the nucleus, which is attached to the other end of the cytoskeleton, and regulate gene expression.^4^^,^^29^

As increased liver stiffness is characteristically observed in ACLD and as its measurement by non-invasive tools has proven to provide accurate risk stratification in this population, we postulated that this mechanical property could *per se* negatively influence hepatic cell biology in cirrhosis. Therefore, in the present study, we investigated whether physiological and increased matrix stiffness, to a level similar to that found in advanced liver fibrosis and in decompensated cirrhosis of the liver, could modulate the phenotype of the main hepatic cell types and represent a possible additional mechanism for the well-known chronic activation of liver cells occurring in cirrhosis. Such a stiffness-induced continued activation of liver cells might contribute both to the progression of liver disease and to prevent its regression after eliminating the cause of liver injury.

In this regard, recent studies suggested that increased matrix stiffness induces the activation of quiescent HSCs into myofibroblast-like cells, whilst hepatocytes isolated from healthy animals display reduced functionality in these conditions.[5], [6], [7]^,^^10^ Therefore, we first validated these previous results in our experimental settings (Fig. S1A and B). LSECs play a major role in the healthy liver and during ACLD.^1^ As the effects of increased matrix stiffness on capillarisation and nitric oxide synthesis in this cell type remain unknown, we additionally characterised the effects that a high stiffness (in the range observed in human cirrhosis) may have on healthy LSECs as to mimic the conditions during progression of ACLD (Fig. S1C). In all these introductory experiments, culture on high stiffness substrates was enough *per se* to modify the expression of activation markers and to impair the morphology and functionality of hepatocytes, HSCs, and LSECs. Therefore, our data are in accordance with the hypothesis that liver cells in an ACLD environment would be continuously receiving activation signals just because of the mechanical properties of the liver.

More importantly, we have herein described for the first time that plating cells from livers of rats with cirrhosis on a matrix with a ‘healthy’ stiffness for just 72 h is able to switch their phenotype back to a more quiescent state. This was observed in all 3 cell types from CCl_4_-exposed livers of rats with cirrhosis and validated in an additional rat model of ACLD (the TAA-injected cirrhosis model) (Fig. 1, Fig. 2, Fig. 3; Fig. S2). Therefore, our data suggest that modulation of the stiffness-related molecular pathway may represent a novel therapeutic strategy to promote regression of fibrosis or cirrhosis. Hence, increased liver stiffness should no longer be considered as a mere consequence of liver fibrosis, but rather an active player in perpetuating hepatocellular dysfunction, liver fibrogenesis, and microvascular dysfunction. We think that this could partly explain previous observations regarding the strong prognostic value of liver stiffness.

Additionally, we hypothesised that activation of liver cells caused by increased stiffness also leads to impaired cell-to-cell communication, further enhancing changes in phenotype. Indeed, previous studies from our group have demonstrated that crosstalk between hepatic cells is determined by their activation state,[30], [31], [32], [33] whilst recent work from Dou *et al.* suggests that HSCs have distinct paracrine secretions in response to varying stiffness.^8^ In the herein presented data, we show for the first time that stiffness regulates the communication between sinusoidal cells of rats with cirrhosis (Fig. 4). Our results suggest that there is some degree of directionality, with stiffness having a greater effect in the communication of HSCs to LSECs than vice versa. These proof-of-concept experiments support future studies aiming at the characterisation of the secretome of liver cell types in response to stiffness, which may lead to the discovery of new therapeutic targets in chronic liver disease.

In addition, we studied whether the ECM stiffness may be an additional factor contributing to the cellular response to antifibrotic strategies. Indeed, the reported beneficial effects of liraglutide on HSC phenotype^21^ were validated in HSCs of rats with cirrhosis cultured in high stiffness conditions, but interestingly these effects were partially lost when HSCs were cultured on low stiffness substrates (Fig. 5). These data are in accordance with previous research in mesenchymal stem cells suggesting that matrix stiffness regulates their response to soluble factors, such as retinoic acid.^34^ Although this requires further in-depth testing, our data open the possibility to consider that the activity of antifibrotic agents may be partly dependent on the ECM stiffness.

After characterising the effects of matrix stiffness on the biology of liver cells during ACLD, we studied the underlying mechanisms. This is relevant for the design of specific strategies targeting the stiffness-related molecular pathways. In that regard, it is worth noting that mechanical forces are transmitted from the ECM to the nucleus through the cytoskeleton (including F-actin and microtubules).^35^ When these forces are strong (as when cells are in a high ECM stiffness environment), the tension applied to the nuclear envelope modifies the shape of the nucleus.^4^^,^^29^ This nuclear deformation in turn has been described as the convergence step of many further downstream mechanisms that regulate gene expression, including conformational changes of proteins and chromatin, changes in protein localisation, and even rupture of nuclear membrane, which together may mediate the observed phenotypical changes.^36^ Indeed, we observed that healthy liver cells (either hepatocytes, HSCs, or LSECs) showed a dramatic degree of nuclear deformation when cultured on high stiffness substrates compared with cells on healthy stiffness (Fig. 6), in some cases even displaying structures resembling chromatin hernias,^37^ although these observations were occasional. Moreover, all the studied liver cell types of rats with cirrhosis exhibiting greatly deformed nuclei when plated on high stiffness cirrhotic-like matrices ameliorated their nuclear morphology to a more spherical shape when cultured on soft matrices, suggesting that the effects of stiffness on nuclear shape are indeed reversible.

One limitation of the present study is the difficulty to set an *in vivo* environment with controlled stiffness. Collagen-coated acrylamide substrates with fine adjustable stiffness may have certain limitations regarding their physical properties^38^ and their 2D architecture. Therefore, although they are well accepted for studying biomechanical forces,^6^^,^^15^ we wanted to validate that nuclear deformation also occurs *in vivo* in cirrhosis by analysing the nuclei of liver cells *in situ* in human liver tissue (Fig. 6D). Interestingly, we observed that cells residing in tissues of patients with cirrhosis displayed marked nuclear deformation compared with those in healthy livers, independently of the aetiology. This deformation, however, was less dramatic than that observed in liver cells cultured on a stiff matrix of patients with cirrhosis, probably caused by complex multidirectional forces associated with a 3D environment.

Nesprins are proteins found in the outer nuclear membrane, which, through their KASH domain, link the cytoskeleton with proteins from the inner nuclear membrane that contain the Sad1/UNC-84 domain.^39^ Therefore, they are critical for the transmission of mechanical tensions from the cytoskeleton to the nucleus. Transfection of human HSCs (both LX-2 and primary HSCs of patients with cirrhosis) with an DN-KASH (unable to interact with the cytoskeleton) ameliorated their nuclear morphology, despite being cultured on high stiffness matrices (Fig. 7A and B). This supports the concept that nuclear morphology is at least partly dependent on mechanical tensions and could be potentially targeted. As transfection of liver cells requires pre-culture and trypsinisation for subsequent seeding on substrate matrices, we used an alternative pharmacological strategy (Cd) to assess the effects of nuclear deformation on cell phenotype. Primary liver cells directly seeded on 30 kPa gels for 72 h were treated with Cd for the last 24 h of culture, which was enough to disrupt their cytoskeleton (Fig. S4) without causing cytotoxicity (Fig. S5), leading to normalisation of their nuclear morphology and amelioration of their activation phenotype (Fig. 7C–E; Fig. S6). These results suggest that targeting tension-mediated nuclear deformation could be a promising strategy against stiffness-induced cell activation and could open the door to future studies aimed at characterising the phenotype of liver cells in response to specific strategies targeting mechanosensing.

Although previous studies have reported nuclear distortion caused by high ECM stiffness,^4^^,^^29^ this is the first time, to our knowledge, that nuclear deformation is described in liver biology in response to changes in ECM stiffness, within the range observed in human disease. This could be relevant because of 2 main implications: first, as a novel molecular pathway with significant effects in ACLD, it can open the path for new therapeutic strategies to counteract the persistent stiffness-induced activation, to be used alone or in combination with existing treatments acting on other mechanisms of cell activation. Second, as stiffness regulates cellular phenotype by altering the mechanical properties of the nucleus (with the described downstream implications in protein and chromatin), it could have consequences not just on a few molecular pathways, but on the whole regulation of gene expression. This underlines that in future studies on novel therapeutic agents, it would be of outmost relevance to consider stiffness conditions mirroring real life, as this could reduce the divergence between the results obtained *in vitro* and *in vivo*. Plastic has an enormous stiffness, over 1 million times of that observed in physiological conditions, a consideration that should temper extrapolations from many previous *in vitro* studies.

**Fig. 8:**
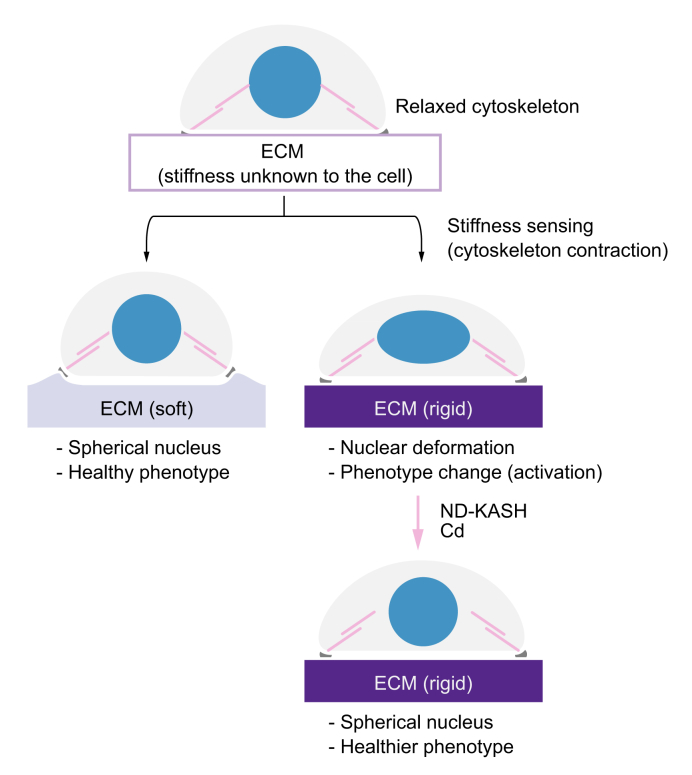
Schematic of the hypothesised mechanism. Cellular sensing of stiff substrates may alter nuclear shape (blue) by increasing tension through the nucleus-cytoskeleton link (magenta) further promoting morphological and phenotypical cell deregulations. Uncoupling the nucleus from the cytoskeleton either by nesprin disconnection (DN-KASH) or pharmacological disruption of the cytoskeleton (Cd) alleviates nuclear tension and recovers healthy cell morphology and phenotype. Cd, cytoskeletal disruptor; ECM, extracellular matrix; DN-KASH, negative dominant nesprin peptide containing a Klarsicht/abnormal nuclear anchorage-1/Syne homology domain.

In conclusion, we describe how liver matrix stiffness, through cytoskeleton-transmitted forces, modulates the phenotype of all 3 main hepatic cell types (hepatocytes, HSCs, and LSECs) in ACLD by altering nuclear shape. Increased matrix stiffness appears to act *per se* as a mechanism perpetuating liver cell activation. Moreover, we report evidence indicating that stiffness could influence cellular crosstalk and the efficacy of drugs with antifibrotic effects, possibly determining different therapeutic windows for each of them. Finally, we provide evidence that interfering with cytoskeleton-dependent tensions prevents and reverts phenotypic changes induced by high stiffness (Fig. 8), suggesting that this may represent a new target for drug therapy.

## Financial support

This project was supported by the 10.13039/501100001711Swiss National Science Foundation (SNF 320030_189252/1), the 10.13039/100008273Novartis Foundation, the Swiss Foundation against Liver Cancer, the 10.13039/501100004837Spanish Ministry of Science and Innovation (10.13039/501100004587Instituto de Salud Carlos III) (FIS PI17/00012), the European Union Fondo Europeo de Desarrollo Regional Funds, ‘una manera de hacer Europa’, and the 10.13039/501100003030Agència de Gestió d'Ajuts Universitaris i de Recerca 2017-SGR-517 and Centres de Recerca de Catalunya programme from the 10.13039/501100002809Generalitat de Catalunya. Part of the work was funded by own research funds of the Hepatology group, Universitätsklinik Viszerale Chirurgie & Medizin, Inselspital, Bern. Centro de Investigación Biomédica en Red en el Área temática de Enfermedades Hepáticas y Digestivas is funded by 10.13039/501100004587Instituto de Salud Carlos III. SG-M has a postdoctoral fellowship from the Stiftung für Leberkrankheiten and Asociación Española Para el Estudio del Hígado. MO-R has a fellowship from the Instituto de Salud Carlos III (iPFIS IFI16/00016). SS has a Juan Rodés Fellowship from the European Association for the Study of the Liver.

## Authors' contributions

SG-M and MO-R designed the research, performed the experiments, analysed the data, and wrote the paper. CW and SS performed experiments and analysed data. IA and JZK performed experiments. CF provided essential materials and revised the paper. PR-C, J-FD, and JB critically revised the paper. AB conceived ideas, obtained funding, and critically revised the paper. JG-S conceived the study, designed and directed the research, wrote the paper, and obtained funding. All authors edited and reviewed the final paper.

## Conflicts of interest

The authors declare no conflicts of interest that pertain to this work.

Please refer to the accompanying ICMJE disclosure forms for further details.

## Supplementary data

Supplementary information.pdf

CTAT table.pdf
