# Supplementary material for: Nuclear deformation mediates liver cell mechanosensing in cirrhosis
Source: JHEP Rep. 2020 Jul 17;2(5):100145. doi: 10.1016/j.jhepr.2020.100145 (PMC7479345; doi:10.1016/j.jhepr.2020.100145)
Supplement: Supplementary information.pdf [file mmc2.pdf]

Nuclear deformation mediates liver cell mechanosensing in cirrhosis

Sergi Guixé-Muntet, Martí Ortega-Ribera, Cong Wang, Sonia Selicean, Ion Andreu, Jenny Z. Kechagia, Constantino Fondevila, Pere Roca-Cusachs, Jean-François Dufour, Jaime Bosch, Annalisa Berzigotti, Jordi Gracia-Sancho

Table of contents

Supplementary methods 2

Supplementary figures 6

Supplementary references 15

Supplementary methods

Isolation of hepatic cells

Briefly, rats were anesthetized with a ketamine + xylazine + midazolam combination (100mg/kg, 10mg/kg, 5mg/kg respectively) and laparotomy was performed. Rat livers were perfused through the portal vein with a modified Hanks' balanced salt solution (HBSS) and digested with 0.015% collagenase A (Roche). For isolation of human cells, liver tissues were perfused with the collagenase solution. In either case, the resulting suspension was filtered through a 100µm cell strainer and cold-centrifuged for 5min at 50x*g*; the pellet contained the hepatocytes while the supernatant was considered the non-parenchymal cell (NPC) fraction. HSC were separated from the rest of NPC with a three-step iodixanol (Sigma) gradient (0%, 11.5% and 17%) and Kupffer cells were removed from the LSEC fraction by unspecific adhesion. Cells were isolated from a minimum of n=4 animals per group and plated in duplicates.

Cell treatments

Liver cells were plated on acrylamide gels and allowed to attach. After two washes with PBS, cirrhotic HSC were treated for 72h with either vehicle (PBS), or liraglutide (50µM, Novo Nordisk Victoza). Simvastatin (10 µM, Calbiochem) was used as positive control for HSC deactivation. Regarding cytoskeleton disruption (Cd) experiments, after 48h of culture, liver cells were treated with a combination of 2µM cytochalasin D (Merck) plus 1µM nocodazole (Merck) or vehicle (dimethylsulfoxide, Sigma) for another 24h (1,2). The total volume for each treatment was prepared in advance in the corresponding medium and added to the plates afterwards in order to eliminate possible pipetting errors.

Preparation of matrix gels with different stiffness

Round coverglasses (12 or 22mm diameter) were treated with a mix of 3-(trimethoxysilyl)propyl methacrylate (Sigma), acetic acid (Panreac) and ethanol (1:1:14 ratio) for 1h in order to activate the glass surface. Subsequently, 9µL/cm^2 of an acrylamide:bis-acrylamide solution (with ammonium persulfate and sodium dodecyl sulfate for polymerization) were added onto the treated coverglasses. New coverglasses were laid on top to extend the mix and the gel was let to polymerize for 1h. After polymerization, the top coverglasses were removed and the surface of the gels was covered with the functionalization solution, containing 50µM HEPES pH=6, 0.004% bis-acrylamide (Bio-Rad), 0.0012% di(trimethylol-propane)tetra-acrylate (Sigma), 0.05% irgacure 2959 (Sigma) and 0.1µg/µL acrylic acid N-hydroxysuccinimide (Sigma). The functionalization of the gels was made effective by 30min exposure to UV light. Finally, gels were rinsed and incubated with the ECM protein (0.1mg/mL rat tail collagen type I). Stiffness was tuned by adjusting the acrylamide:bis-acrylamide ratios (3). Before use, gels were placed inside culture plates, sterilized with UV light and equilibrated with the corresponding culture media for 2h.

Bright field morphology analysis

Cell morphology was quantified by image analysis as follows. From each image, regions containing cells were analyzed using three different image texture descriptors (color histograms, rgb; local binary patterns, lbp; grey level co-occurrence matrices, glcm) using the scikit-image library for Python 3 (4). The resulting histograms were fitted into a Random Forest classifier in order to determine the most important bin for

each of the feature histograms (rgb, lbp, glcm) and labelled as Descriptor 1, Descriptor 2 and Descriptor 3 for simplicity. Thus, each image was described as a 3D data point describing its texture and subsequently represented in a 3D scatter plot. Data points from each experimental condition were represented in a different color and a minimum volume ellipsoid was represented around them for an easier visualization. The graph orientation was determined by visual exploration of the 3D volume and the most relevant hyperplane was selected.

Immunofluorescence and confocal microscopy

Cells were fixed directly on the polyacrylamide gels with 4% paraformaldehyde for 10 min, rinsed with PBS and permeabilized with 0.1% triton X-100 (Sigma) for 5 min. Unspecific binding sites were blocked for 30 min with 1% bovine serum albumin (BSA) in PBS and subsequently incubated overnight at 4ºC with the primary antibody against α-smooth muscle actin (α-SMA, 1:200, Sigma) or phalloidin (1:1000, Sigma). For α-SMA staining, the secondary antibody, conjugated with Alexa Fluor 488 (1:300, ThermoFisher), was incubated for 1h at room temperature in combination with DAPI (3ng/ml, ThermoFisher). Preparations were then mounted using Fluoromount-G (Bionova científica) and dried over-night. Six images per preparation and channel (bright field; blue, 405nm; green, 488nm or red, 545nm) were obtained with a spectral confocal microscope (Leica TCS-SP5). For α-SMA determination, images were taken at 200X magnification while for nuclei characterization images were taken at 630X magnification. Image analyses of fluorescence and area were performed using the ImageJ (Fiji) software (5). The same settings were used for gathering and

quantification of all images from the same experiment. Intensities were normalized to the number of cells per field.

Analysis of nuclear deformation

Samples were fixed and stained with DAPI as described above. Using the confocal microscope, 97 consecutive images per field were taken along the z-axis every 0.21µm in order to capture the whole nuclei height. The 3D reconstruction of the nuclear volume was performed using ImageJ (Fiji). Digital nuclei reconstructions were then thresholded and rotated along the x-axis and the aspect ratio of the nuclear silhouette was measured every 30º (aspect ratio = long diagonal / short diagonal; aspect ratio of a perfect circle = 1). The rotation with the highest aspect ratio was used as a measure of deformation for each nucleus (e.g. the measure of deformation for a perfect sphere = 1 since all its rotations display a circular silhouette. On the other hand, a coin-shaped nucleus will have a higher deformation value, since some of its rotations' shapes are not circular but elliptical). All aspect ratio measurements were obtained using the ImageJ's *Analyze Particles* plugin. Nuclear deformation was analyzed in at least 100 nuclei per experimental condition.

Real-time polymerase chain reaction (RT-PCR)

mRNA was isolated and purified using the RNeasy Mini and Micro Kits (Qiagen) according to manufacturer's instructions. RNA was quantified using a Nanodrop spectrophotometer and retro-transcription to cDNA was performed using the Quantitect Reverse Transcription Kit (Qiagen). RT-PCR was performed using the

Taqman Fast Universal PCR Master Mix (Applied Biosystems) and predesigned Taqman probes (HNF4α, Rn04339144_m1; albumin, Rn00592480_m1; α-SMA, Rn01759928_g1; collagen I, Rn01463848_m1; Hhip, Rn01431433_m1; Lrat, Rn00574091_m1; laminin b1, Rn01473698_m1; eNOS, Rn02132634_s1 and Pdgfrb, Rn01491838_m1) in a 7500 Fast Real-time PCR System (Applied Biosystems). CT values were normalized to those of 18S (Hs99999901_s1) and expressed as relative changes versus the control (ΔΔCT method).

LDH release

LDH was assessed in the supernatant of cells transfected or treated with Cd using standard methods at the Hospital Clínic of Barcelona's CORE laboratory.

Supplementary figures

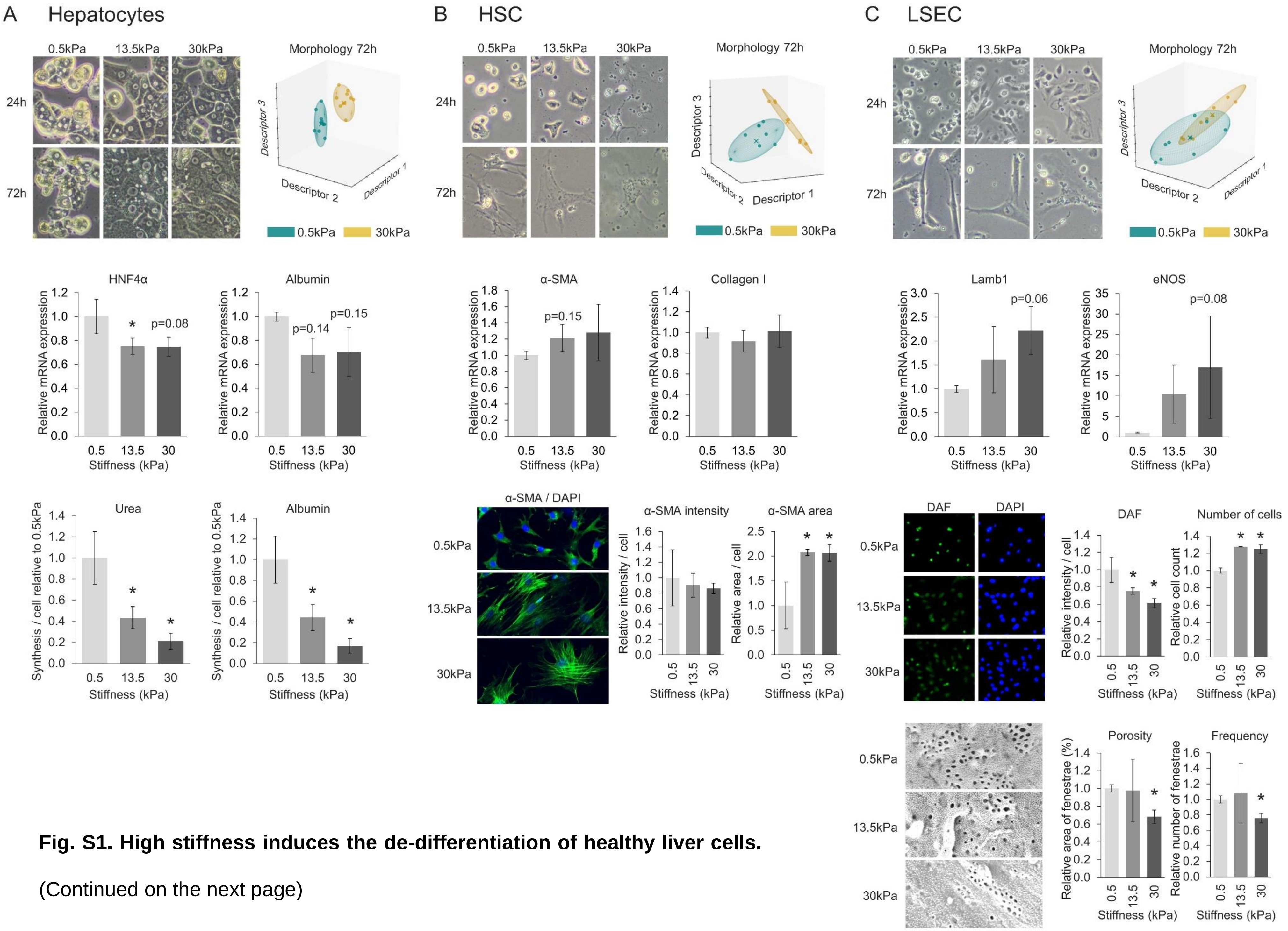

Fig. S1. High stiffness induces the de-differentiation of healthy liver cells.

(Continued on the next page)

Fig. S1. High stiffness induces the de-differentiation of healthy liver cells.

Hepatocytes (A), HSC (B) and LSEC (C) isolated from healthy rats were cultured for 72h on polyacrylamide gels with increasing stiffness. *Top,* bright field images and corresponding morphology quantification. Descriptors 1, 2 and 3 correspond to grey level co-occurrence matrices, color histogram and local binary patterns, respectively. *Middle*, mRNA expression of phenotype markers after 72h culture. *Bottom*, phenotype markers at the post-transcriptional level: urea and albumin released by hepatocytes during the last 24h of culture, immunofluorescence of α-SMA (in green) in HSC and corresponding quantification of intensity and area (DAPI in blue), and nitric oxide synthesis (DAF, green; DAPI, blue) and fenestrae in LSEC. Data derive from n=4 independent experiments and are expressed as mean ± standard error of the mean. For each experiment, sample distributions were assessed for normality (Kolmogorov-Smirnov test) and homoscedasticity (Levene's test). The ANOVA was performed in homoscedastic groups following a normal distribution, followed by the Tuckey's post-hoc test. Otherwise, the non-parametric Kluskal-Wallis was performed, followed by the Mann-Whitney U test. *p-value < 0.05 vs 0.5kPa. p-value > 0.2 if not specified.

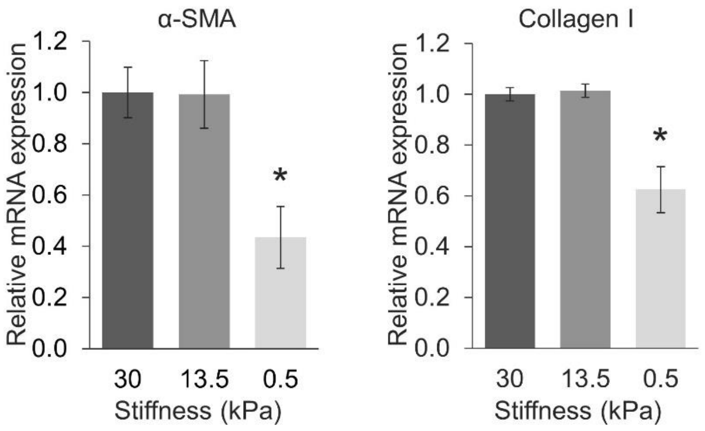

Fig. S2. Healthy stiffness promotes the improvement of TAA-cirrhotic HSC.

Expression of the HSC activation markers α-SMA and collagen I in primary HSC isolated from TAA-cirrhotic rats and cultured for 72h on polyacrylamide gels with decreasing stiffness. Data derive from n=3 independent experiments and are expressed as mean ± standard error of the mean. For each experiment, sample distributions were assessed for normality (Kolmogorov-Smirnov test) and homoscedasticity (Levene's test). The ANOVA was performed in homoscedastic groups following a normal distribution, followed by the Tuckey's post-hoc test. Otherwise, the non-parametric Kluskal-Wallis was performed, followed by the Mann-Whitney U test. *p-value < 0.05 vs 30kPa. p-value > 0.2 if not specified.

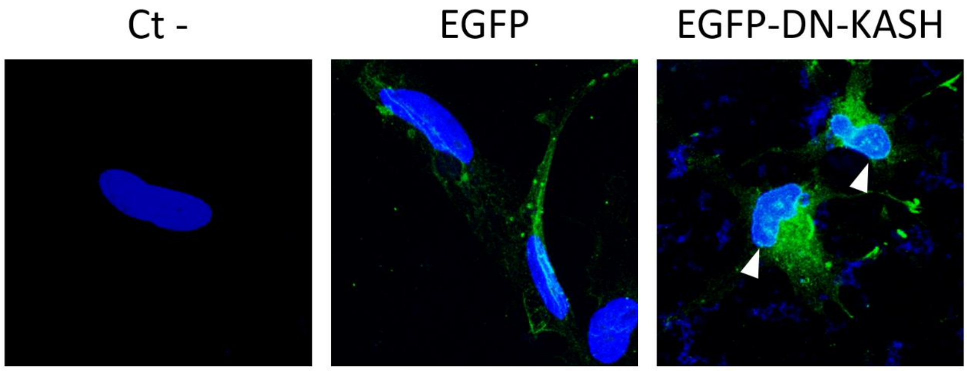

Fig. S3. Verification of DN-KASH transfection. Transfection of EGFP or the fusion protein DN-KASH-EGFP was confirmed in HSC by green fluorescence. White arrows indicate DN-KASH localizing to the nucleus.

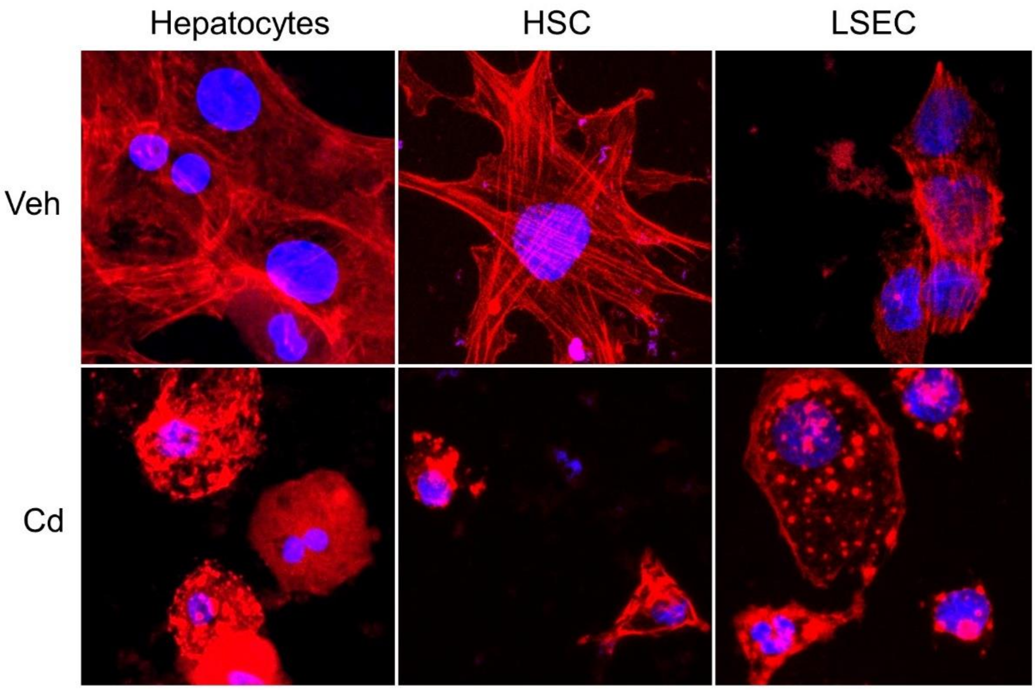

Fig. S4. Effectiveness of the cytoskeleton disruptors.

Phalloidin staining (red) in liver cells cultured for 72h on 30kPa matrices and treated with vehicle or cytoskeleton disruptors (Cd) for the last 24h. Nuclei were stained with DAPI (blue). Images were taken at 630X magnification.

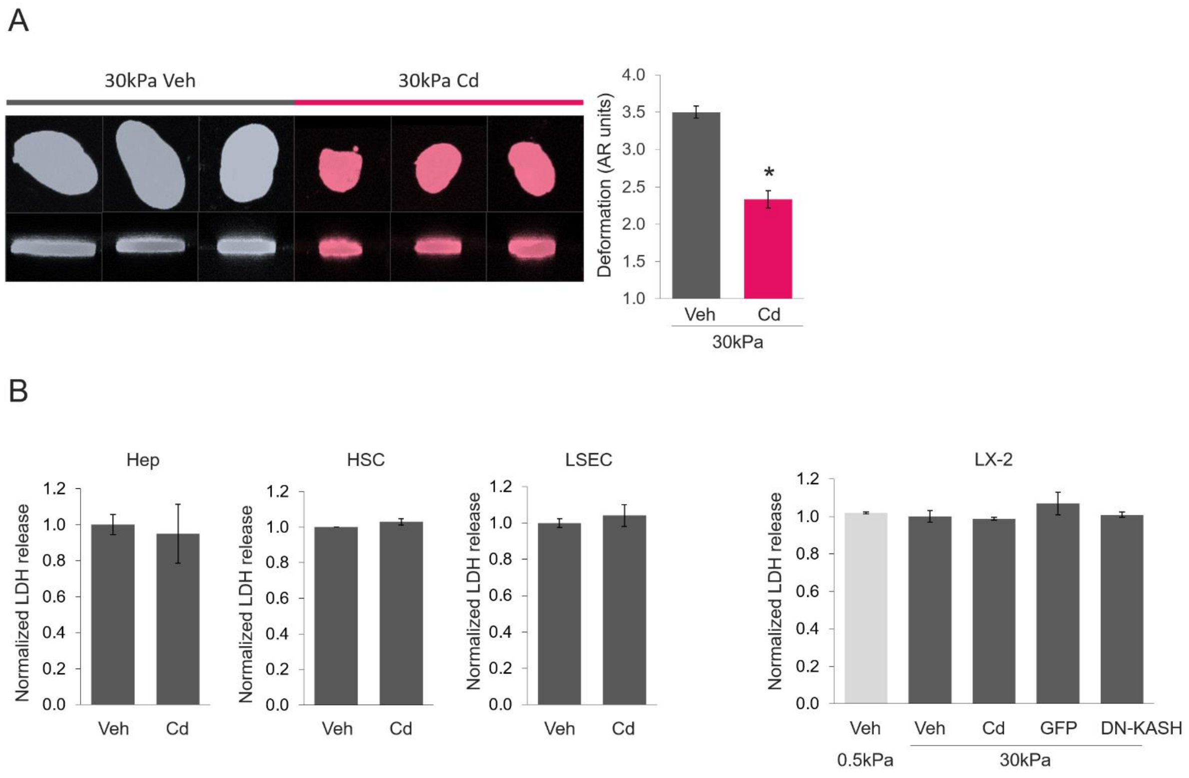

Fig. S5. Toxicity assessment in nuclei-cytoskeleton uncoupling approaches.

LX-2 were cultured for 72h on 30 kPa matrices and treated with cytoskeleton disruptors (Cd) or vehicle (Veh) for the last 24h. Nuclear morphology was analyzed by confocal microscopy (A). Toxicity derived from Cd treatment or plasmid transfection was assessed in primary isolated cells (B left panels) and LX-2 (B right panel) as LDH release to the culture media. Data derive from n=3 independent experiments and are expressed as mean ± standard error of the mean. For each experiment, sample distributions were assessed for normality (Kolmogorov-Smirnov test) and homoscedasticity (Levene's test). For experiments with two groups, these were compared with the Student's T-test. For multiple comparisons, the ANOVA was performed in homoscedastic groups following a normal distribution. Otherwise, the non-parametric Kluskal-Wallis was performed. *p-value < 0.05 vs Veh. p-value > 0.2 if not specified.

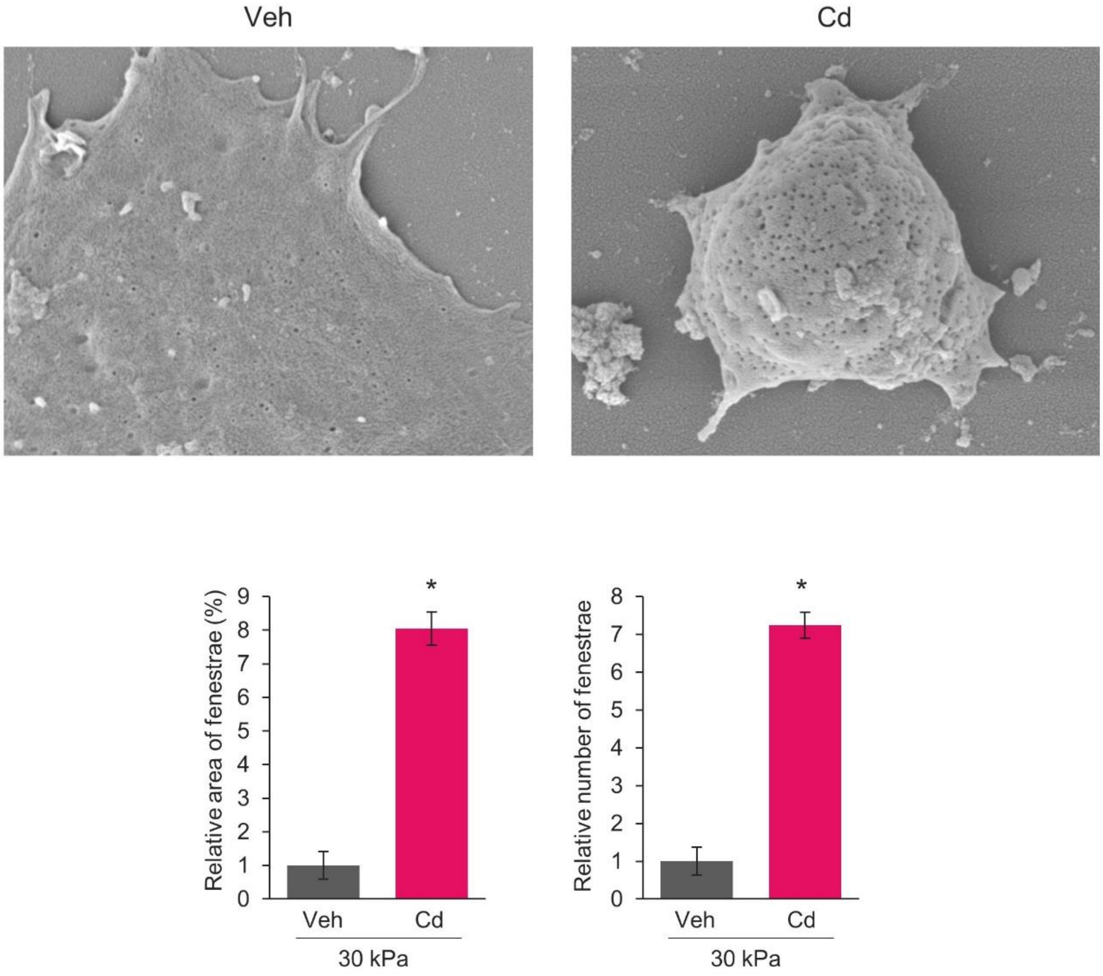

Fig. S6. LSEC fenestrae in response to cytoskeleton disruptors. LSEC were seeded on rigid matrices (30kPa) and treated with cytoskeleton disruptors (Cd) or vehicle (Veh) for the last 24h. Representative scanning electron microscopy images (5000X) and fenestrae porosity and frequency quantifications are shown. Data derive from n=3 independent experiments and are expressed as mean ± standard error of the mean. Groups were compared with the Student's T-test *p-value < 0.05 vs Veh.

Supplementary Video 1. 3D morphology of the nuclei from hepatocytes in response to stiffness.

Nuclei from hepatocytes cultured for 72h on 0.5kPa and 30kPa gels (A) or on 30kPa in the presence of vehicle (Veh) or cytoskeleton disruptors (Cd) (B). More than 50 nuclei were analyzed for each experimental condition. Images were taken at 630X magnification. N=3 independent experiments.

Supplementary references

1. Marchesi C, Dall'Asta V, Rotoli BM, Bianchi MG, Maggini C, Gazzola GC, et al. Chlorpromazine, clozapine and olanzapine inhibit anionic amino acid transport in cultured human fibroblasts. Amino Acids. 2006;31:93–9.
2. **Kim D-H, Li B**, Si F, Phillip JM, Wirtz D, Sun SX. Volume regulation and shape bifurcation in the cell nucleus. J. Cell Sci. 2015;128:3375–3385.
3. **Dou C, Liu Z, Tu K**, Zhang H, Chen C, Yaqoob U, et al. P300 Acetyltransferase Mediates Stiffness-Induced Activation of Hepatic Stellate Cells Into Tumor-promoting Myofibroblasts. Gastroenterology. 2018;154:2209–2221.
4. Van Der Walt S, Schönberger JL, Nunez-Iglesias J, Boulogne F, Warner JD, Yager N, et al. Scikit-image: Image processing in python. PeerJ. 2014;2014:e453.
5. Schindelin J, Arganda-Carreras I, Frise E, Kaynig V, Longair M, Pietzsch T, et al. Fiji: an open-source platform for biological-image analysis. Nat. Methods. 2012;9:676–682.
