# Supplementary material for: Nuclear deformation mediates liver cell mechanosensing in cirrhosis
Source: JHEP Rep. 2020 Jul 17;2(5):100145. doi: 10.1016/j.jhepr.2020.100145 (PMC7479345; doi:10.1016/j.jhepr.2020.100145)
Supplement: CTAT table.pdf [file mmc3.pdf]

JHEP|Reports

JHEP Reports

CTAT methods

Tables for a "Complete, Transparent, Accurate and Timely account" (CTAT) are now mandatory for all revised submissions. The aim is to enhance the reproducibility of methods.

- Only include the parts relevant to your study
- Refer to the CTAT in the main text as 'Supplementary CTAT Table'
- Do not add subheadings
- Add as many rows as needed to include all information
- Only include one item per row

If the CTAT form is not relevant to your study, please outline the reasons why:

1.1 Antibodies

Name	Citation	Supplier	Cat no.	Clone no.
Anti-αSMA	Direkze NC et al. Cancer Res. 2004 ;64(23):8492-5.	Sigma	A2547	1A4
Phalloidin	Tavares S et al. Nat Commun. 2017 8:15237. doi: 10.1038/ncomms15237.	Sigma	P1951	from *Amanita phalloides*

1.2 Cell lines

Name	Citation	Supplier	Cat no.	Passage no.	Authentication test method
LX-2	Xu L et al. Gut. 2005;54(1):142-51.	Kindly provided by Dr. Bataller.	n/a	12-15	

1.3 Organisms

Name	Citation	Supplier	Strain	Sex	Age	Overall n number
Rat		Charles River	Wistar Han	Male	4-20 weeks	20
Rat		Janvier	Sprague - Dawley	Male	16 weeks	8

1.4 Sequence based reagents

Name	Sequence	Supplier
Taqman probes used for qPCR	HNF4α, Rn04339144_m1<br>albumin, Rn00592480_m1<br>α-SMA, Rn01759928_g1<br>collagen I, Rn01463848_m1	ThermoFisher Scientific

	laminin b1, Rn01473698_m1<br>eNOS, Rn02132634_s1<br>Pdgfrb, Rn01491838_m1<br>18S Hs99999901_s1	

1.5 Biological samples

Description	Source	Identifier

1.6 Deposited data

Name of repository	Identifier	Link

1.7 Software

Software name	Manufacturer	Version
Fiji (Image J)	Open contribution	Version 1.8.0_112
Graphpad Prism	Graphpad Software Inc.	Version 8.0.2
Phython3	Python	Version 3.6.7

1.8 Other (e.g. drugs, proteins, vectors etc.)

Liraglutide	Novo Nordisk	
SImvastatin	Calbiochem	567021
Cytochalasin D	Merck	C8273
Nocodazole	Merck	M1404
Diaminofluorescein diacetate (DAF-FM)	Life Technologies	D-23844
EGFP-empty or EGFP-Nesprin1-KASH plasmid	Kindly provided by Catherine Shanahan	n/a. Described in Zhang et al. 2001 J. Cell Sci.

1.9 Please provide the details of the corresponding methods author for the manuscript:

Jordi Gracia-Sancho, PhD. Hepatology, Department of Biomedical Research, University of Bern. Murtenstrasse 35, Maurice E. Müller-Haus, F821a, 3008 Bern. email: jordi.gracia@dbmr.unibe.ch

2.0 Please confirm for randomised controlled trials all versions of the clinical protocol are included in the submission. These will be published online as supplementary information.

n/a
